# Microbiome-Derived Metabolites in Multiple Sclerosis: Mechanisms of Immune Regulation and Therapeutic Potential: A Literature Review

**DOI:** 10.7759/cureus.117645

**Published:** 2026-10-08

**Authors:** Maysaa N Amin

**Affiliations:** 1 Microbiology/Immunology, California Institute of Behavioral Neurosciences and Psychology, Fairfield, USA

**Keywords:** gut-brain axis, gut microbiota, immune regulation, microbiome-derived metabolites, multiple sclerosis, probiotics, short-chain fatty acids

## Abstract

Multiple sclerosis (MS) is a chronic autoimmune neuroinflammatory disease characterized by immune dysregulation, demyelination, and neurodegeneration of the central nervous system (CNS). Increasing evidence suggests that gut microbiota and microbiome-derived metabolites play important roles in the pathogenesis and progression of MS by modulating the gut-brain-immune axis. This narrative review examines the possible immunomodulatory effects of microbiome-derived metabolites in MS, with particular emphasis on short-chain fatty acids (SCFAs), tryptophan-derived metabolites, and polyamines, and evaluates their potential therapeutic implications. This review was conducted using the most recent available studies relevant to the topic. The search included PubMed, Google Scholar, and PMC databases. Eligible studies focused on randomized controlled trials, systematic reviews, meta-analyses, observational studies, reviews, and other publications that examined the role of microbiota and its metabolites in MS.

The reviewed evidence indicates that MS-associated dysbiosis is accompanied by altered microbial metabolite production, disruption of regulatory T cell (Treg)/T helper 17 (Th17) balance, impaired barrier integrity, and increased neuroinflammation. SCFAs, tryptophan-derived metabolites, and polyamines demonstrate immunoregulatory effects through modulation of T-cell responses, cytokine signaling, and inflammatory pathways. Microbiota-targeted interventions, including probiotics, prebiotics, dietary modulation, and metabolite supplementation, have shown promising immunological and clinical effects. However, methodological heterogeneity, small cohorts, variable interventions, and insufficient long-term clinical validation limit definitive conclusions regarding therapeutic efficacy. Future large-scale longitudinal and multi-omics studies are crucial for clarifying the underlying mechanisms and unlocking microbiota-targeted strategies to manage MS more effectively.

## Introduction and background

Multiple sclerosis (MS) is a long-lasting autoimmune inflammatory disorder that affects the central nervous system (CNS). This condition is characterized by dysregulation of peripheral immunity and infiltration of immune cells into the CNS, resulting in demyelination and damage to axons, ultimately leading to neurodegeneration [1]. Upon activation, naive CD4+ T cells differentiate into Treg cells, Th17 cells, and T helper type 1 (Th1) and T helper type 2 (Th2) helper subsets. Significantly, the breakdown of immunological tolerance in MS is primarily driven by increased pro-inflammatory activity of Th17 cells and decreased immunosuppressive capacity of Treg cells [2]. Th17 cells are recognized for their production of the pro-inflammatory cytokine IL-17 and their migration to the CNS during the active phase of the disease [3,4]. Simultaneously, patients with MS exhibit dysregulation of CD4+ Treg cells, characterized by a significant decrease in their suppressive capacity [5,6].

Despite the fact that the fundamental mechanisms of MS are still not fully understood, studies have indicated that autoreactive T cells can penetrate a compromised blood-brain barrier (BBB) and activate particular inflammatory pathways in the CNS, resulting in neuroinflammatory cascades [7]. Although disease-modifying therapies have substantially improved the management of MS, they do not fully address the complex environmental and metabolic factors that may influence disease activity. Consequently, increasing attention has been directed toward the gut microbiome as a potential modulator of immune and neuroinflammatory pathways [8].

Gut microbiota and gut-brain axis

The CNS regulates gastrointestinal physiological and immunological functions, while the gut influences CNS development and function through the intestinal microbiota and its metabolites, immune cells, gut hormones, and the vagus nerve [9,10]. The gut microbiota communicates with the CNS through simultaneous and interrelated pathways: neuronal, endocrine, and immune pathways [11,12].

The gut microbiota can also influence neuronal and neuroendocrine signaling through microbial products, including neurotransmitter-related compounds [13,14]. Conversely, the CNS can influence gut function through neural, hormonal, and immune mechanisms [11,12]. The bidirectional communication between the gastrointestinal tract (GIT) and the CNS is referred to as the gut-brain axis. Immune cells in the gut and CNS also contribute to gut-brain communication, while interactions between the microbiota and innate and adaptive immune systems influence both microbial composition and intestinal immune function [15].

Gut dysbiosis generally refers to alterations in microbial composition, diversity, or functional activity that may be associated with disruption of host-microbiome homeostasis. Dysbiosis may alter microbial metabolic activity and intestinal barrier integrity, potentially contributing to systemic inflammation [16]. Changes in the circulating metabolome may have a functional role in the pathophysiology of MS in addition to being possible indicators of disease risk and prognosis [17]. It has been proposed that the intestinal environment, specifically chemicals produced by the microbiota, promotes myelin-specific Th17 pathogenicity in the gut during experimental autoimmune encephalomyelitis (EAE) and may be linked to disease severity in persons with MS (PwMS) [18]. This bidirectional communication between the GIT and the CNS is illustrated in Figure 1.

**Figure 1:**
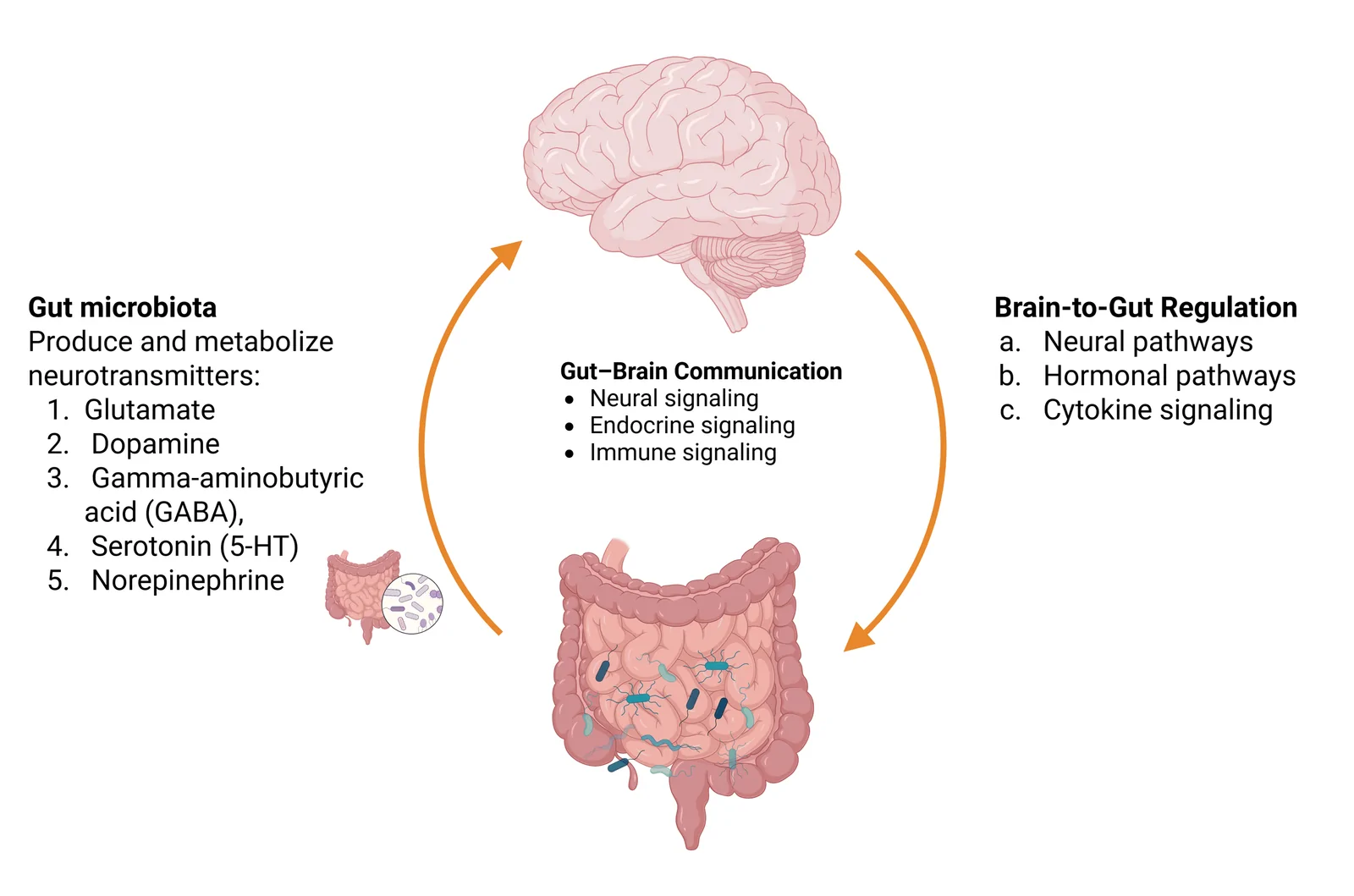
Schematic representation of the gut-brain axis showing bidirectional communication between the GIT and the CNS through neural, endocrine, immune, and microbial metabolite signaling pathways Figure created by the author using BioRender.com

Microbiome-Derived Metabolites in Immune Regulation

Microbiome-derived metabolites are important mediators of the gut-brain axis and contribute to energy metabolism, intercellular signaling, immune regulation, and neuroimmune communication [19]. Short-chain fatty acids (SCFAs), including acetate, propionate, and butyrate, are among the most thoroughly researched microbiome-derived metabolites that are involved in gut-brain communication. Produced through bacterial fermentation of dietary fibers, SCFAs support immunological homeostasis, maintenance of intestinal and BBB integrity, and modulation of neuroinflammatory responses [20,21]. Additionally, alterations in SCFA profiles, including reduced circulating butyric acid, have been associated with changes in intestinal barrier permeability and inflammation in patients with MS, supporting a potential role for SCFAs in gut-immune regulation [22]. SCFAs are produced by a number of bacteria, including *Butyricimonas*, *Faecalibacterium*, and *Clostridium* clusters IV and XIVa, which are reduced in MS patients. *Akkermansia*, on the other hand, has been shown to rise in MS [23,24].

In addition to SCFAs, tryptophan-derived metabolites represent another important class of microbiota-mediated signaling molecules involved in gut-brain communication. Gut microorganisms can metabolize the essential amino acid tryptophan into indole-containing compounds, including indole-3-lactic acid (ILA), indole-3-acetic acid (IAA), and indole-3-aldehyde (IAld) [25]. Through pathways including aryl hydrocarbon receptor (AhR) signaling, these metabolites may influence inflammatory T-cell responses and cytokine production in MS [26,27]. Alterations in tryptophan-derived metabolite profiles have also been associated with MS disease activity.

Polyamines represent another important group of microbiome-associated metabolites that may influence neuroinflammation through immune-cell regulation. They include putrescine, spermidine, and spermine. These metabolites are produced through the L-arginine metabolic pathway and may originate from either the host or the gut microbiota. Polyamines, particularly spermidine, may alleviate neuroinflammation by influencing the activity of immune cells and fostering the polarization of anti-inflammatory T cells [28].

Table 1 summarizes the major microbiome-derived metabolites discussed in this review, including SCFAs, tryptophan-derived metabolites, and polyamines, together with their principal bacterial producers, biosynthetic pathways, and reported immunoregulatory effects.

**Table 1:** The metabolites of the microbiome and their immunomodulatory actions on MS AhR: aryl hydrocarbon receptor; BBB: blood-brain barrier; CNS: central nervous system; EAE: experimental autoimmune encephalomyelitis; HDAC: histone deacetylase; IL-22: interleukin-22; IAld: indole-3-aldehyde; IPA: indole-3-propionic acid; MS: multiple sclerosis; SCFAs: short-chain fatty acids; Th17: T helper 17 cells; Treg: regulatory T cells.

Microbiome-derived metabolite(s)	Product	Produced Bacteria	Pathways	Effects in Multiple Sclerosis	References
SCFAs	Butyrate	Faecalibacterium prausnitzii	Fermentation of dietary fiber through the acetyl-CoA and butyryl-CoA pathways	Enhances Treg differentiation, suppresses Th17 responses, reduces neuroinflammation, and BBB disruption	[29,30]
SCFAs	Propionate	*Bacteroides fragilis* and *Veillonella* spp.	Succinate pathway and acrylate pathway during carbohydrate fermentation	Restores immune tolerance, increases anti-inflammatory cytokines, and improves Treg/Th17 balance	[30,31]
SCFAs	Acetate	*Akkermansia muciniphila* and *Bifidobacterium* spp.	Mucin fermentation and carbohydrate metabolism through pyruvate conversion (*A. muciniphila*) and the bifid shunt (fructose-6-phosphate phosphoketolase pathway) (*Bifidobacterium* spp.)	Regulates microglial maturation and decreases inflammatory signaling in CNS tissues	[30, 32]
SCFAs	Butyrate, acetate	Roseburia inulinivorans	Fiber fermentation with HDAC inhibitory activity mediated by SCFAs	Epigenetic regulation of immune cells via histone deacetylase inhibition, promoting anti-inflammatory responses	[30]
SCFAs	Butyrate	*Butyricimonas* spp.	Anaerobic fermentation of resistant starches and polysaccharides	Reduced abundance observed in MS-associated dysbiosis; linked to lower SCFA availability and enhanced inflammation	[30, 33]
Tryptophan metabolites	Indole-3-propionic acid (IPA)	Clostridium sporogenes	Tryptophan metabolism through the indole pyruvate pathway	Activates AhR, suppresses astrocyte-mediated inflammation, and promotes neuroprotection	[29]
Tryptophan metabolites	Indole derivatives	Lactobacillus reuteri	Tryptophanase-mediated indole production and AhR ligand synthesis	Enhances mucosal immune homeostasis, limits pro-inflammatory CNS immune infiltration	[29, 34]
Tryptophan metabolites	Kynurenine-related metabolites	*Bacteroides* spp.	Kynurenine pathway activation during dysbiosis-associated inflammation	Increased neurotoxic kynurenine metabolites contribute to demyelination and disease progression	[34]
Tryptophan metabolites	Indole-3-aldehyde (IAld)	Peptostreptococcus russellii	Conversion of dietary tryptophan into AhR agonists	Regulates IL-22 production and intestinal immune tolerance, affecting gut-brain signaling	[29]
Polyamines	Spermidine	Bifidobacterium adolescentis	Arginine decarboxylase and ornithine decarboxylase pathways	Promotes autophagy, suppresses inflammatory cytokines, supports oligodendrocyte survival	[30]
Polyamines	Putrescine	Escherichia coli	Ornithine decarboxylation pathway	Modulates macrophage activation and influences neuroimmune signaling in EAE/MS models	[30]
Polyamines	Spermine	*Lactobacillus* spp.	Methionine salvage and polyamine biosynthesis pathways	Associated with modulation of immune and neurological processes	[35]
Polyamines	Spermidine and spermine	Enterococcus faecalis	Arginine deiminase and polyamine biosynthesis pathways	Contributes to epithelial barrier integrity and immunomodulation in gut-brain axis signaling	[30]

Therapeutic Implications

Given the potential role of microbiome-derived metabolites in immune regulation, strategies aimed at modifying the gut microbiota and its metabolic activity have attracted increasing attention in MS research. Dietary strategies incorporating bioactive components have also been investigated for their potential to support cognitive health and influence neuroinflammatory processes in neurodegenerative diseases [36]. Due to their ability to trigger anti-inflammatory reactions by affecting the immune system and gut microbiota, probiotics, prebiotics, and postbiotics are becoming more widely acknowledged as important elements in the management of MS. Nevertheless, only a limited number of studies have explored the specific roles of these factors in MS. Both prebiotics and postbiotics contribute positively to gut and immune system health, albeit through distinct mechanisms [37].

Postbiotics can enhance host-specific physiological functions, regulatory mechanisms, and metabolic and/or behavioral responses associated with the activity of the host's indigenous microbiota, including SCFAs, enzymes, cell surface proteins, and vitamins [38]. In contrast to postbiotics, which exert their effects through microbial-derived bioactive compounds, prebiotics consist of dietary fibers or foods that enhance the growth and/or activity of beneficial native probiotic bacteria [39]. In essence, prebiotics affect the composition and functionality of the gut microbiota, enabling probiotic bacteria to endure and multiply as they pass through the upper GIT, with limited disruption from other microorganisms [40].

Prebiotics have positive effects on the gut microbial flora, while probiotics are live microorganisms that provide health benefits to the host when taken in sufficient quantities. Probiotics have been subject to extensive studies as potential treatments for diseases related to microbiota due to their diverse immunoregulatory roles, such as regulating immune response, blocking pathogens, regulating microbial balance in the intestines, and maintaining gastrointestinal barrier integrity [41]. Based on earlier clinical studies, the consumption of multi-strain probiotics may result in a notable decrease in serum inflammatory biomarkers, including C-reactive protein (CRP), tumor necrosis factor alpha (TNF-α), and interferon gamma (IFN-γ) [42]. These findings indicate the potential benefits of manipulating the gut microbiome to maintain immune balance and reduce neuroinflammation in patients with MS.

However, the exact nature of the immunomodulation caused by the metabolites produced by the microbiome is not well known, particularly regarding how they influence immune pathways and whether these mechanisms can be translated into therapeutic strategies, even though there is growing evidence, particularly in terms of the roles played by metabolites such as SCFAs and derivatives of tryptophan. Therefore, this review examines the immunomodulatory roles of microbiome-derived metabolites in MS, with particular emphasis on SCFAs, tryptophan-derived metabolites, and polyamines, and evaluates their potential therapeutic implications. Additionally, this review highlights the current limitations in the literature and points out some areas that need future research.

## Review

Methods

This review was conducted in accordance with the Scale for the Assessment of Narrative Review Articles (SANRA) guideline [43]. The studies chosen aimed to explore the immunoregulatory role of metabolites derived from the microbiome in MS, as well as their potential therapeutic applications.

Literature Search Strategy

A structured literature search was conducted using PubMed, PubMed Central (PMC), and Google Scholar to identify publications relevant to microbiome-derived metabolites, immune regulation, and multiple sclerosis (MS). The primary search concepts included “multiple sclerosis,” “microbiome-derived metabolites,” “microbiota,” “brain-gut axis,” “immune regulation,” “immune therapy,” “immunomodulation,” and “therapeutic strategies.” PubMed searches were conducted using relevant keywords and Medical Subject Headings (MeSH), combined using Boolean operators such as “AND” and “OR.” The final search across all databases was performed in June 2026.

The PubMed search strategy was based on the following terms and MeSH headings:

((Multiple Sclerosis OR "Multiple Sclerosis/diagnosis"[Majr] OR "Multiple Sclerosis/diagnostic imaging"[Majr] OR "Multiple Sclerosis/diet therapy"[Majr] OR "Multiple Sclerosis/drug therapy"[Majr] OR "Multiple Sclerosis/immunology"[Majr] OR "Multiple Sclerosis/therapy"[Majr]) AND (Microbiome-Derived Metabolites OR microbiota OR brain-gut axis OR "Brain-Gut Axis/drug effects"[Majr] OR "Brain-Gut Axis/genetics"[Majr] OR "Brain-Gut Axis/immunology"[Majr]) AND (Immune Regulation OR Therapeutic Strategies OR Immune therapy OR Immunomodulation OR "Immunomodulation/drug effects"[Majr] OR "Immunomodulation/genetics"[Majr] OR "Immunomodulation/immunology"[Majr]))

Google Scholar was used as a supplementary source to identify potentially relevant publications not identified through the primary database searches.

Eligibility Criteria and Study Selection

Eligible publications were English-language, full-text, open-access articles relevant to the immunoregulatory role of microbiome-derived metabolites in MS and their potential therapeutic applications. The review considered randomized controlled trials involving human participants, systematic reviews, meta-analyses, literature reviews, case-control studies, cohort studies, observational studies, and cross-sectional studies. Mini-reviews, conference abstracts, and animal-only studies were excluded from the formal synthesis of evidence, although animal studies were considered when necessary to provide relevant biological or mechanistic context.

The study selection process involved an initial screening of titles and abstracts for relevance, followed by assessment of the full text of potentially eligible publications against the predefined eligibility criteria and the objectives of the review.

Data Extraction and Narrative Synthesis

Relevant information regarding microbiome-derived metabolites, their microbial sources, mechanisms of immune regulation, MS-related findings, and potential therapeutic implications was extracted from the selected publications and synthesized narratively according to the major metabolite groups examined in this review.

Discussion

Gut microbiota and their products have been found to play a critical role in the immunopathogenesis of MS. Experimental and clinical studies suggest that gut dysbiosis may lead to immune dysregulation through alterations in microbial metabolite production, ultimately disrupting Treg/Th17 balance, neuroinflammation, and the BBB. Despite substantial evidence indicating the involvement of the gut microbiome in MS, the exact mechanisms by which gut microbes influence immunity in MS remain unknown [44]. Collectively, the reviewed studies indicate that microbiome-derived metabolites act as key intermediaries between gut dysbiosis and immune dysfunction in patients with MS. Through modulation of T cells, cytokine production, antigen-presenting cells, and gut-brain immunological transmission, changes in microbial composition and metabolic activity seem to affect the state of balance between pro-inflammatory and regulatory immune pathways. 

Gut Microbiota Dysbiosis and Treg/Th17 Immune Imbalance in MS

Gut dysbiosis in MS is consistently associated with the disruption of Treg/Th17 immune homeostasis, characterized by impaired regulatory immune responses and enhanced pro-inflammatory activity [14]. Reduced abundance of immunomodulatory bacterial genera, including *Clostridia*, *Bacteroides*, *Prevotella*, and *Faecalibacterium*, has been linked to impaired Foxp3+ Tregs differentiation and increased Th1/Th17-mediated neuroinflammation. Several microbial products, including polysaccharide A and lipid 654, may contribute to immune tolerance by activating tolerogenic dendritic cells, suppressing inflammatory macrophage activity, and inducing anti-inflammatory cytokines, such as IL-10 [45,46].

Human microbiome studies further support the relationship between gut dysbiosis and systemic immune activation in patients with MS. A six-month longitudinal multi-omics study involving 49 adults (24 untreated relapsing-remitting MS patients and 25 healthy controls) demonstrated significant alterations in the gut microbiota composition, blood metabolome profiles, and immune cell populations in MS patients. Specifically, reduced abundance of beneficial immunomodulatory bacteria, including *Bifidobacterium longum*, *Clostridium leptum*, *Faecalibacterium prausnitzii*, *Prevotella*, and members of the *Lachnospiraceae* family, was observed in MS patients compared with healthy controls [47]. These microbial alterations were accompanied by increased peripheral pro-inflammatory immune signatures, including elevated T-bet+ effector T cells and Th17 cell populations, suggesting a mechanistic link between microbial depletion and immune dysregulation in patients with MS [47].

Similar findings were reported in a Danish case-control study involving 148 MS patients and 148 matched healthy controls, where 61 bacterial species were found to differ significantly between groups, with several MS-associated taxa positively correlating with inflammatory biomarkers, including CRP, leukocyte counts, and expression of IL17A and IL6. Moreover, plasma cytokines associated with disease activity, including IL-17A, IL-22, TNF-α, and interferon beta (IFN-β), correlated with bacterial species enriched in treatment-naïve patients with active MS [48]. In contrast, non-disease-active patients demonstrated increased abundance of anti-inflammatory bacterial species such as *Faecalibacterium prausnitzii* and *Gordonibacter urolithinfaciens*, whose metabolites, including butyrate and urolithin, have demonstrated protective immunomodulatory effects in experimental models of MS [48]. Collectively, these findings support the concept that altered gut microbial ecology may contribute to MS-associated immune activation via metabolite-mediated regulation of inflammatory pathways [48].

Notably, administration of *Prevotella histicola*, a commensal bacterium found in the gut, modulates systemic immune responses, including downregulating pro-inflammatory Th1/Th17 responses and increasing CD4+FoxP3+ Treg cells, tolerogenic DCs, and suppressive macrophages, to suppress CNS inflammatory and demyelinating disease as effectively as the MS treatments glatiramer acetate and interferon beta [48]. These findings highlight the potential of microbiota-based therapeutic approaches aimed at restoring immune tolerance and controlling neuroinflammatory pathways in MS [49].

Nevertheless, the following drawbacks exist, including small sample size, different approaches in the sequencing and analyzing of the microbiome, diverse dietetic and geographic characteristics of the patients involved in research, and the absence of clinical evidence gathered over a long period. Furthermore, it remains unknown whether gut dysbiosis is a cause or consequence of the development of the disease. Thus, future controlled clinical interventions and longitudinal research are needed to establish causality and to target specific bacterial strains with the greatest therapeutic potential in order to establish a viable supplementary strategy to current MS treatments [48]. Figure 2 illustrates these findings.

**Figure 2:**
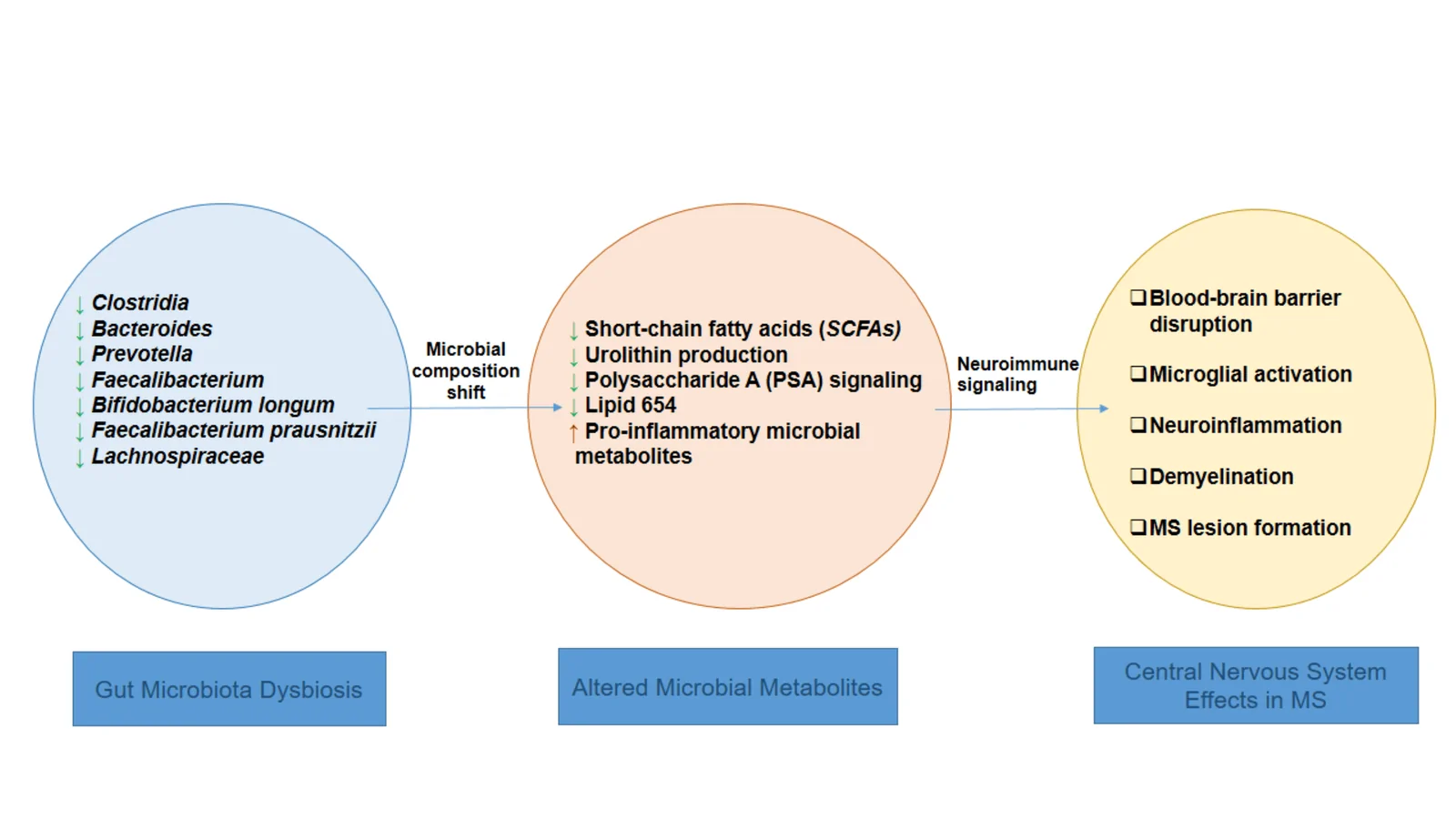
A proposed pathogenic pathway in MS from gut dysbiosis to neuroinflammation Figure created by the author using PowerPoint

The Metabolite-Immune Interplay (SCFA, Tryptophan, Polyamine)

Among the most thoroughly researched microbiota-derived metabolites involved in immune modulation in MS patients are SCFAs, including acetate, propionate, and butyrate. SCFAs promote the differentiation of FoxP3+ Tregs through GPR41/GPR43 signaling pathways while suppressing pro-inflammatory Th1 and Th17 responses. In addition, SCFAs reduce the production of inflammatory cytokines, including IFN-γ, TNF-α, IL-1β, and IL-6, while enhancing anti-inflammatory mediators such as IL-10 and TGF-β, thereby contributing to the maintenance of immune homeostasis [50-52]. 

Combined findings from MS patients and the murine EAE model indicate that altered microbiota composition and functionality may contribute to CNS-specific autoimmunity through an imbalance of T cell subpopulations, including Th1, Th2, Th17, and Treg cells. These results imply that substantial changes in the gut microbiome's microbial taxonomy and the ensuing shortfall in SCFA production in the gut are at least largely linked to decreasing the threshold for the onset and aggravation of MS [53,54]. One experimental study revealed that patients with MS had lower total concentrations of SCFA than healthy controls (HCs) (p-value = 0.022). When comparing MS to HCs, acetate levels were substantially lower (p-value = 0.021). Compared to HCs, MS had considerably lower acetate/butyrate and acetate/propionate + butyrate ratios (p-value = 0.005/0.01). The findings showed a negative correlation between acetate levels and the pro-inflammatory cytokine IFN-γ in healthy controls, indicating an inverse relationship between acetate and inflammation [55].

Clinical research has consistently shown that MS patients have lower amounts of SCFAs and fewer SCFA-producing bacteria than healthy controls. Reduced fecal and serum concentrations of acetate, propionate, and butyrate have been associated with altered gut microbial composition, increased Th17-related inflammation, and impaired Treg responses [23,56-58]. Apart from the above, an observational case-control study was conducted to account for confounding variables such as dietary habits, geographical location, and genetic factors by comparing MS patients with healthy individuals residing in the same household. This study found significant changes in the gut microbiome's composition and function in MS patients. Untreated MS patients had a significant reduction in *Faecalibacterium prausnitzii*, a member of the phylum *Firmicutes*, compared to those receiving treatment with interferon-beta. *Faecalibacterium prausnitzii* is a crucial butyrate producer and possesses anti-inflammatory characteristics, including the inhibition of IL-8 production, upregulation of Treg cells, and suppression of expression through the nuclear factor κB pathway [24].

Importantly, both experimental and clinical findings suggest that restoration of SCFA production through dietary modulation, probiotics, or direct metabolite supplementation may partially correct the Treg/Th17 imbalance and reduce inflammatory activity in MS. Among SCFAs, propionate has received particular attention because supplementation has been associated with immunological changes involving regulatory and pro-inflammatory T-cell populations in MS. However, larger randomized clinical trials are required before propionate supplementation can be considered an established therapeutic intervention [59,60]. Despite these promising findings, current evidence remains heterogeneous due to differences in dietary habits, geographical backgrounds, disease-modifying therapies, and microbiome sequencing methodologies across studies.

While SCFAs are the microbiota-derived metabolites involved in immune regulation, other microbial metabolic pathways also contribute to neuroimmune homeostasis in MS. In particular, tryptophan-derived microbial metabolites have emerged as important regulators of neuroimmune signaling in MS through AhR activation. AhR is a cytoplasmic receptor that regulates immune responses and autoimmunity through DCs, T cells, macrophages, and natural killer (NK) cells [61]. According to recent reports, chronic EAE was alleviated when AhR was knocked down, which also increased the production of microbial metabolites, including SCFAs and bile acids [61]. 

Clinical evidence further supports the immunoregulatory role of this signaling pathway. An exploratory cohort study enrolled people with MS and healthy controls, who were administered a lactobacilli-rich probiotic (Vivomixx®) for two or six weeks, with a follow-up six weeks after termination. The results showed that probiotic supplementation enriched with *Lactobacillus* strains increased circulating Treg populations, elevated IL-10 expression, and enhanced tryptophan metabolite IAA production in patients with MS [62]. These immunological effects were AhR-dependent, highlighting the therapeutic relevance of the microbiota-tryptophan-AhR axis in neuroinflammatory diseases. An AhR inhibitor was added to prevent this effect on the cells. The findings link probiotic-induced increased synthesis of the gut microbial tryptophan metabolite IAA to enhanced regulatory T cell responses and IL-10 production in people with MS, suggesting the control of the AhR pathway as a strategy [62]. Apart from the above, the study showed that even under B-cell depleting therapy, lactobacilli supplementation may restore a regulatory immunological phenotype in patients with MS, suggesting promise as an adjuvant therapy in addition to current disease-modifying treatments [62].

Furthermore, polyamines, including spermidine, spermine, and putrescine, represent an emerging class of microbiota-associated metabolites involved in immune regulation and are abundant within the GIT [28]. An experimental study proposed that spermidine acts as an immunosuppressive metabolite promoting gut tolerance by favoring Treg differentiation through autophagy induction. The findings demonstrated that spermidine significantly promotes the differentiation of naive CD4+ T cells into Foxp3+ Tregs while reducing proinflammatory IL-17-producing Th17 cells in vitro. Supplementation with L-arginine (a precursor metabolized to spermidine) also enhanced Treg frequencies and serum spermidine levels, suggesting microbiota involvement in polyamine metabolism. The research uncovers spermidine as a key modulator of T cell fate via autophagy, contributing to immune homeostasis in the gut and protection against inflammatory disease [28]. Although evidence in MS remains limited compared with SCFAs and tryptophan metabolites, these findings highlight polyamine metabolism as a promising area for future therapeutic investigation.

These results highlight the need for extensive clinical trials to further explore the metabolite-immune interaction and its potential preventive and therapeutic applications. They also offer important insights into the immune defense mechanisms linked to gut microbiota diversity and its metabolites in MS.

Therapeutic Translation (Prebiotics, Probiotics)

Given the central role of microbiota-derived metabolites in immune regulation, modulation of gut microbial composition has emerged as a potential adjunctive therapeutic strategy in MS. Through the restoration of metabolite-producing bacterial populations, the enhancement of SCFA and tryptophan-derived metabolite production, and the subsequent correction of dysregulated immune responses linked to neuroinflammation, prebiotics, probiotics, and dietary treatments may have an impact on disease activity.

Evidence from observational and case-control studies supports this association. A Canadian pediatric case-control study involving 95 participants reported that higher adherence to an alternative Mediterranean diet (aMED) was associated with a significantly lower risk of pediatric-onset MS, with each 1-point increase in the aMED score corresponding to a 34% reduction in MS odds (adjusted odds ratio (ORadj) 0.66, 95% confidence interval (CI) 0.47-0.90, p-value = 0.011). Increased fiber intake was also associated with reduced MS risk and correlated with enrichment of beneficial bacterial taxa, including *Ruminococcaceae* and *Clostridiales* species, which are known contributors to SCFA production and intestinal immune regulation [63]. Moreover, cross-sectional studies and pilot clinical trials demonstrated that higher Mediterranean diet adherence was associated with lower fatigue, depression, disability, and disease severity scores among MS patients [64-66]. Furthermore, reduced dietary fiber intake has consistently been observed in MS populations compared with healthy controls [67]. Mechanistically, these dietary patterns may influence neuroinflammation through modulation of gut microbial metabolism, promotion of SCFA-producing bacteria, and suppression of Th17-associated inflammatory pathways.

In contrast, diets characterized by higher meat consumption have been associated with increased circulating Th17-related immune markers and reduced abundance of fiber-metabolizing bacteria such as *Bacteroides thetaiotaomicron* [47]. Findings from the International Multiple Sclerosis Microbiome Study (iMSMS) further demonstrated overlap between diet-associated microbial taxa and microbiota signatures linked to MS susceptibility [24], supporting the concept that dietary habits influence disease progression through microbiota-mediated immune mechanisms [63].

Nevertheless, emerging evidence suggests that responses to prebiotic interventions may depend on the individual microbial composition and metabolic capacity of the host. A recent study demonstrated reduced abundance of β-fructan-fermenting bacteria, including *Bifidobacterium *species, in pediatric-onset patients with MS, suggesting impaired microbial fermentation of specific fibers. Consequently, excessive intake of certain fermentable fibers may not uniformly provide benefit and could potentially worsen inflammatory responses in susceptible individuals. These findings highlight the need for personalized microbiome-based nutritional approaches rather than universal dietary recommendations in MS management [68].

In addition to dietary and prebiotic approaches, probiotics have also been investigated as microbiota-targeted interventions in MS. Evidence from randomized controlled trials and meta-analyses generally supports the immunomodulatory potential of probiotics in MS. A meta-analysis of 28 randomized controlled trials involving more than 2000 patients with autoimmune diseases demonstrated that microbiome-targeted therapies, including probiotics and synbiotics, significantly reduced the pro-inflammatory cytokine TNF-α in MS patients (standardized mean difference (SMD) −1.41, 95% CI −1.99 to −0.82; p-value < 0.001). These interventions were also associated with increased total antioxidant capacity (TAC), potentially mediated through SCFA-induced activation of antioxidant pathways such as Nrf2 signaling [69].

Consistent with these findings, another systematic review and meta-analysis reported that probiotic supplementation reduced expression of pro-inflammatory cytokines, including IFN-γ and TNF-α, while increasing IL-10 levels and regulatory T cell activity in MS patients. These immunological effects were accompanied by beneficial alterations in gut microbiota composition, suggesting that probiotics may exert therapeutic effects through modulation of the microbiota-metabolite-immune axis [70]. Clinical trials further support these observations. A six-month randomized clinical study involving 70 MS patients demonstrated that multi-strain probiotic supplementation containing *Lactobacillus*, *Bifidobacterium*, and *Streptococcus thermophilus* strains significantly reduced IL-6 levels, while increasing brain-derived neurotrophic factor (BDNF) in addition to improving mental health parameters [42].

Similarly, in a small randomized clinical trial involving 40 patients with MS, four months of *Saccharomyces boulardii* supplementation was associated with improvements in pain, fatigue, and quality-of-life scores in patients with relapsing-remitting MS. Crucially, the probiotic group had significantly lower levels of total TAC and high-sensitivity C-reactive protein (hs-CRP) compared to the placebo group (p-values < 0.001 and 0.004, respectively). However, the small sample size and short follow-up limit conclusions regarding long-term clinical efficacy [71]. Figure 3 illustrates the potential effects of dietary patterns on the gut microbiota, microbiota-derived metabolites, and the clinical outcomes in MS.

**Figure 3:**
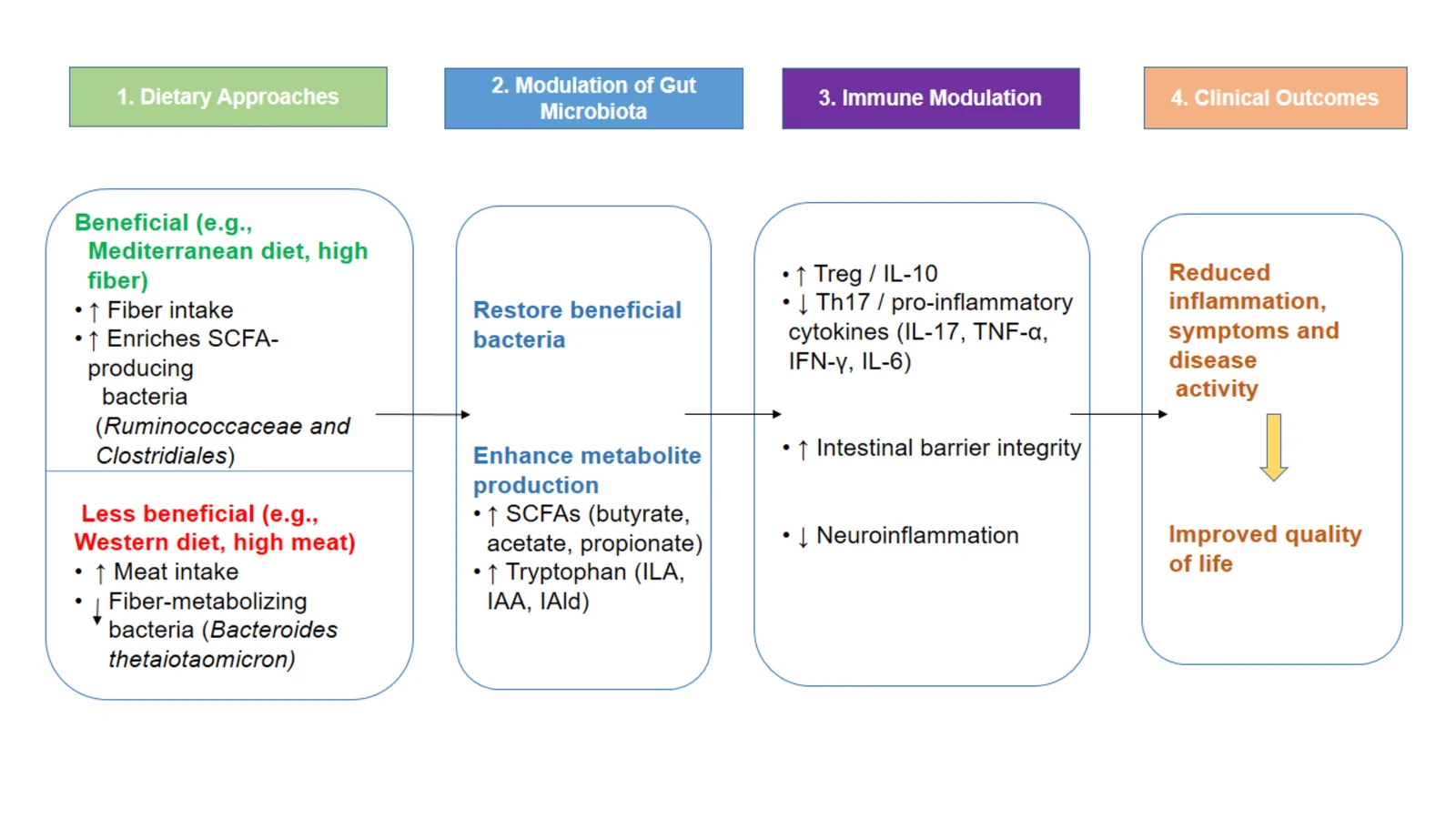
Therapeutic translation of the gut microbiota-metabolite-immune axis in MS SCFAs: short-chain fatty acids; ILA: indole-3-lactic acid; IAA: indole-3-acetic acid; IAld: indole-3-aldehyde; Treg: regulatory T cells; Th17: T helper 17 cells; TNF-α: tumor necrosis factor alpha; IFN-γ: interferon gamma. Figure created by the author using PowerPoint

Despite these promising findings, the existing data on probiotics remain limited by substantial heterogeneity in probiotic strains, treatment duration, disease subtype classification, and concurrent disease-modifying therapies across studies. In addition, most clinical trials remain short-term and primarily evaluate immunological or symptomatic outcomes rather than long-term neurodegeneration or disease progression. Therefore, larger multi-center longitudinal studies integrating microbiome, metabolomic, and immunological profiling are required to identify the most effective probiotic formulations and establish microbiota-targeted therapies as clinically validated adjunctive interventions in MS.

Limitations and Future Considerations

Although this review was conducted in accordance with SANRA guidelines and focuses on evidence from randomized controlled trials, systematic reviews, meta-analyses, and observational studies, several limitations should be considered. The review emphasized recent advances in microbiome and metabolomic research; however, the selection of predominantly recent evidence may have limited the inclusion of some earlier foundational mechanistic studies. Considerable methodological heterogeneity across the included studies also limits direct comparison and interpretation of findings. Variations in microbiome sequencing methods, metabolomic profiling techniques, probiotic formulations, intervention duration, dietary assessment, and outcome reporting may contribute to inconsistent results. In addition, geographic, dietary, environmental, and ethnic differences between study populations may influence gut microbiota composition and reduce generalizability. Another important limitation is that many studies included relatively small sample sizes and short follow-up periods, reducing statistical power and limiting assessment of long-term therapeutic efficacy and safety. Furthermore, much of the current evidence remains observational and associative, and therefore cannot determine whether dysbiosis and altered metabolite profiles contribute to MS pathogenesis or arise secondarily from disease, treatment, diet, lifestyle, or other environmental factors.

To bridge the gap between associative findings and clinical application, translational research should prioritize four strategic domains. First, longitudinal metabolomic and immune profiling may help develop early-warning systems and identify stage-specific biomarkers of MS activity and progression. Recent studies indicate that blood metabolomic signatures can distinguish disease phenotypes and predict clinical outcomes, supporting investigation of metabolic signatures as tools for disease staging and therapeutic-window definition [72,73]. Second, integrated microbiome, metabolomic, transcriptomic, and single-cell analyses combined with network pharmacology and machine-learning approaches may help investigate potentially causal mechanisms underlying dysbiotic associations and identify actionable metabolite-receptor pathways [74]. Particular attention should be given to SCFA signaling through GPR43 (FFA2) and GPR41 (FFA3), together with the tryptophan-AhR pathway, while recognizing that these pathways may exert context-dependent effects in neuroinflammation [75,76]. Third, neuroprotective metabolite-delivery strategies warrant investigation to address progressive neuroaxonal injury that remains insufficiently controlled by anti-inflammatory therapies. Propionic acid and butyric acid have promoted neurite recovery in human MS-derived neuronal models, while recent clinical trial evidence supports further investigation of propionic acid as an adjunctive metabolite-based intervention [77,78]. Future studies could therefore evaluate engineered nanocarriers for controlled delivery of selected microbiota-derived metabolites and their effects on neuroinflammation and axonal preservation. Fourth, next-generation biodelivery systems, including engineered hydrogels, could facilitate localized and controlled delivery of microbiota-derived therapeutics in response to intestinal microenvironmental cues [79]. Their applicability to MS, however, requires rigorous preclinical validation and clinical evaluation.

## Conclusions

The evidence reviewed highlights an important role for gut microbiota and microbiome-derived metabolites in the immunopathogenesis of MS, supporting a mechanistic link between gut dysbiosis and immune dysregulation. MS patients consistently demonstrate reduced abundance of beneficial immunomodulatory bacteria and altered microbial metabolic activity, which may contribute to disruption of Treg/Th17 immune homeostasis and promotion of neuroinflammatory pathways.

SCFAs, metabolites of tryptophan, and polyamines derived from gut microbiota have emerged as potentially important immune mediators in MS through cytokine secretion, induction of regulatory T cells, and reduction of pro-inflammatory immune responses. Clinical studies further suggest that microbiota-targeted interventions, including probiotics, prebiotics, and dietary modulation, may beneficially influence immune responses and clinical outcomes in MS, highlighting their potential as adjunctive therapeutic strategies. However, methodological heterogeneity, small cohort sizes, and inadequate long-term clinical validation continue to limit existing findings. Furthermore, the precise causal relationship between gut dysbiosis, altered microbial metabolites, and MS progression remains to be fully elucidated. To clarify underlying mechanisms and determine the therapeutic potential of microbiota-targeted therapies in MS management, future large-scale longitudinal and multi-omics research incorporating microbiome, immunological, and clinical data is necessary.
